# Developing a synergistic rate-retarding polymeric implant for controlling monoclonal antibody delivery in minimally invasive glaucoma surgery

**DOI:** 10.1016/j.ijbiomac.2024.132655

**Published:** 2024-06

**Authors:** Mengqi Qin, Jinyuan Luo, Brihitejas Patel, Kai Xin Thong, Samar Latefa, Daniel Shao, Alexander Tanner, Cynthia Yu-Wai-Man

**Affiliations:** Faculty of Life Sciences & Medicine, King's College London, London SE1 1UL, UK

**Keywords:** Glaucoma, MIGS, Monoclonal antibody, Ocular drug delivery, Polymer

## Abstract

Monoclonal antibodies (mAbs) have garnered substantial attention within the field of ophthalmology and can be used to suppress scar formation after minimally invasive glaucoma surgeries. Here, by controlling mAb passive diffusion, we developed a polymeric, rate-controlling membrane reservoir loaded with poly(lactic-co-glycolic acid) microspheres to deliver mAb for several weeks. Different parameters were tested to ensure that the microspheres achieved a good quality characteristic, and our results showed that 1 %*W*/*V* emulsifier with 5 %W/V NaCl achieved mAb-loaded microspheres with the highest stability, encapsulation efficiency and minimal burst release. Then, we fabricated and compared 10 types of microporous films based on polylactic acid (PLA), polycaprolactone (PCL), and polyethylene glycol (PEG). Our results revealed distinct pore characteristics and degradation patterns in different films due to varying polymer properties, and all the polymeric film formulations showed good biocompatibility in both human trabecular meshwork cells and human conjunctival fibroblasts. Finally, the optimized microspheres were loaded into the reservoir-type polymeric implant assembled by microporous membranes with different surface coating modifications. The implant formulation, which was fabricated by 60 PCL: 40 PEG (3 %*W*/*V*) polymer with 0.1 %W/V poly(lactic-*co*-glycolic acid) barrier, exerted the best drug release profile that can sustained release mAb (83.6 %) for 4 weeks.

## Graphical abstract

**Figure:**
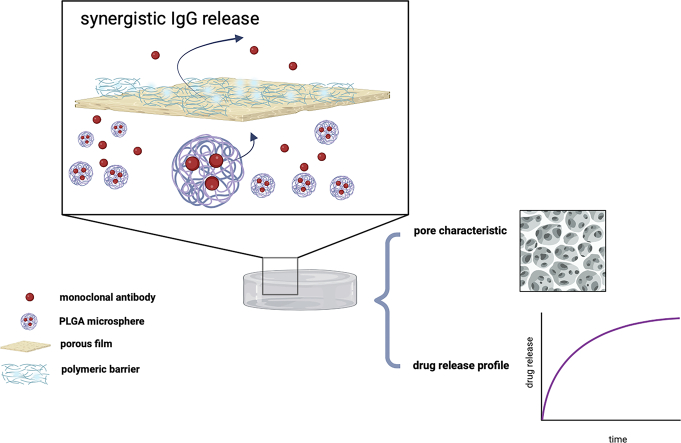

## 1 Introduction

Glaucoma is the leading cause of irreversible blindness in the world. In 2040, global estimates of glaucoma cases are forecasted to reach 111.8 million [1]. Minimally invasive glaucoma surgeries (MIGS) represent an intraocular pressure (IOP)-lowering procedure that employs micro-incision. Their popularity has surged globally, reflecting a significant increase in adoption rates [2]. However, post-operative fibrosis can lead to stent obstruction and is the critical determinant of the long-term surgical success in MIGS [3,4].

Monoclonal antibodies (mAbs) have been developed for clinical use since the late 1980s [5]. Due to its advantages of low toxicity, high efficacy and targeting ability, mAb therapy has become increasingly popular [6]. mAb therapy has also been extensively used in ophthalmology, for example, the chimeric anti-TNF-α mAbs, infliximab and adalimumab, have exhibited superior clinical efficacy compared to traditional steroids and immunosuppressants, while demonstrating a limited side effect profile to control uveitis and other inflammatory eye diseases [7,8]. Given that repeated intraocular injections of drugs can lead to poor compliance and the risk of complications, many intracameral devices like Allergan's Durysta™ and Glaukos' iDose™, which provide sustained drug release, have been developed and have either entered or are poised to enter the market [9]. Unlike other biologics, mAbs are sensitive to various harsh conditions [10]. For example, IgG would become irreversibly denatured and completely lose its functional activity at a temperature higher than 65 °C [11]. Hence, conventional scaffold fabrication methods, such as directly blending drugs and biomaterials followed by the formation of these homogenous mixtures into scaffold structures using 3D printing or molding techniques, may subject IgG to heat treatment or harsh solvents, potentially compromising the antibody's bioactivity.

Encapsulating therapeutic mAbs in biodegradable poly(lactic-*co*-glycolic acid) (PLGA) microparticles is an effective way to control the antibody's sustained release [[12], [13], [14], [15]]. PLGA is a copolymer formed through the copolymerization of lactic and glycolic acid monomers [16]. Depending on the lactic/glycolic acid ratio, PLGA can exhibit different physicochemical properties. It has been reported that 50:50 PLGA-based microparticles not only exhibit high drug loading and releasing efficacy, but also show great biocompatibility in the rabbit eye [17,18]. In addition, microparticles composed of 50:50 PLGA can completely degrade after three months and release degradation products within a limited period, ensuring its safety as an ocular drug delivery system [19]. Hence, 50:50 PLGA microparticles are among the promising candidates for ocular drug delivery formulations.

However, the high concentration of microparticles can result in blurred vision during intraocular administration due to a light scattering effect [20], and directly injecting bevacizumab-PLGA microspheres intravitreally can also lead to a severe inflammatory response [21]. Therefore, confining drug-loaded PLGA microparticles within an implant proves to be a safer approach for enhancing ocular drug delivery. The rate-controlling polymeric film has been widely applied in the pharmaceutical manufacturing field and is the main component in the drug delivery implant [[22], [23], [24], [25], [26]]. The drug release rate from the microporous films depends on the pore size, porosity and tortuosity of the film [27]. Blending two or more polymers by solvent casting and particulate leaching technology is an effective way to modify the intrinsic porous structure, crystallinity and mechanical properties of the polymeric film [28]. Polylactic acid (PLA) and polycaprolactone (PCL), both biodegradable synthetic aliphatic polyesters, have received FDA approval for tissue engineering applications [29,30]. In contrast to hydrophilic natural polymers like polysaccharides and various animal proteins, their slow degradation and limited hydrolytic properties, both *in vitro* and *in vivo*, make them ideal candidates for use in rate-controlling polymeric films for extended drug retention periods. However, to maximally optimize the polymeric films for improved drug residence time, the selection of the most suitable polymer mixture and the precise control of the fabrication parameters, such as polymer concentration and blending ratio, are further required.

In this study, our focus was to address the crucial need for prolonged therapeutic mAb retention to prevent the postoperative fibrotic process following MIGS. To achieve this goal, we developed a novel PLGA microparticle-loaded intraocular implant capable of sustained mAb release over 4 weeks. Detailed experiments involving adjusting emulsifier concentration and osmotic pressure during the fabrication were conducted to ensure the microspheres exhibited high encapsulation efficiency, minimal burst release, and sustained stability over time. Furthermore, our investigation progressed to the development of polymeric films utilizing PLA and PCL as primary retarding polymers, with PEG serving as a porogen. By adjusting the composition of the polymeric film, we observed corresponding changes in thickness, pore characterization, pore formation mechanism, and degradation properties. Using a thorough analysis, we examined the drug release profiles of implants comprised of various microporous membranes. This multifaceted approach provides valuable insight into the optimization of drug delivery systems, but also holds significant clinical promise in advancing therapeutic strategies for the management of postoperative fibrosis in MIGS patients, thereby enhancing the surgical outcomes and patient care.

## 2 Materials and methods

### 2.1 Materials

IgG (I5381), penicillin-streptomycin (P4333), D-(+)-trehalose dihydrate (T9531), poly(vinyl alcohol) (PVA) (Mw = 13,000–23,000 Da, 87–89 % hydrolysed) (363170), dimethyl sulfoxide (DMSO) (D4540) and dichloromethane (DCM) (75-09-2) were purchased from Merck Sigma, UK. Phosphate buffered saline (14190-094) was bought from Avantor. Dulbecco's modified Eagle's medium (DMEM) (41966-029), l-glutamine (25030-024), and fetal calf serum (FCS) (10270-106) were purchased from Gibco, Thermo Scientific. Polylactic acid (PLA) (Mw = 30,000 Da) (Sc-255440), polycaprolactone (PCL) (Mw = 50,000 Da) (25090-100), polyethylene glycol (PEG) 3350 (25322-68-3), and poly(lactic-*co*-glycolic acid) (PLGA) (50:50) (Mw = 10,000–20,000 Da) (34346-01-5) were purchased from Insight Biotechnology, Generon, Spectrum Chemical Mfg. Corp, and Cambridge Bioscience, respectively. The physiochemical properties of PLA, PCL and PLGA used in this study have been summarized in Table S1.

### 2.2 IgG-loaded PLGA microsphere preparation and optimization of the microparticle formulations

The IgG-loaded PLGA microspheres were prepared by water-in-oil-water (W_1_/O/W_2_) double emulsion method. To prepare the primary W_1_/O emulsion, 20 μL of 2.5 % *W*/*V* IgG solution with 10 % *W*/*V* trehalose [31] were emulsified in a PLGA solution (10 mg PLGA in 500 μL DCM) by sonication (30 s, 65 W). W_2_ solution (10 % *W*/*V* trehalose) is composed of 2 % W/V, 1 % W/V and 0.5 % W/V PVA and with or without 5 % W/V NaCl. The obtained W_1_/O solutions were added dropwise into 2 mL of W_2_ solution and sonicated for 30 s at 65 W. Then, 3.5 mL of W_2_ solution were added into W_1_/O/W_2_ solution and stirred at room temperature for 2 h to evaporate the organic solvent. The PLGA microspheres were then collected and washed three times with deionized water by centrifuging at 13,000 rpm for 1 h each time. The microparticles were pre-frozen in a −80 °C freezer and then dried using a lyophilizer machine. The lyophilized microparticles were stored in a −20 °C freezer until usage.

In order to achieve microparticles with high drug loading, *in vitro* stability, and drug releasing profile, we assessed the effects of the manufacturing conditions (concentrations of the emulsifier and salt, respectively, in the external water phase) on the particle size, polydispersity index, encapsulation efficiency and drug releasing profile. The parameters involved in the PLGA microsphere fabrication are summarized in Table 1.

**Table 1:** The composition of the PLGA microsphere formulations.

Formulation	PLGA (mg)	IgG (W/V)	W_2_ (W/V)	NaCl (W/V)
MP1	10	5 %	2 % PVA	0 %
MP2	10	5 %	1 % PVA	0 %
MP3	10	5 %	0.5 % PVA	0 %
MP4	10	5 %	1 % PVA	5 %
MP5	10	5 %	0.5 % PVA	5 %
Plain MP4	10	0 %	1 % PVA	5 %
Plain MP5	10	0 %	0.5 % PVA	5 %

### 2.3 Physicochemical characterization of the PLGA microsphere

#### 2.3.1 Particle size, polydispersity index and *in vitro* stability of the PLGA microparticle

The test formulations were suspended into 1 mL deionized water and the suspension was added in the disposable folded capillary cell (Malvern). The particle size and polydispersity index were measured by dynamic light scattering (Zetasizer Pro, Malvern). Each sample was tested in triplicates.

The *in vitro* stability of the microsphere was detected by suspending the samples in deionized water for 1, 2, 3 and 4 weeks. At each timepoint, 1 mL of each sample was collected and measured using the Zetasizer Pro to compare the changes in particle size and PDI.

#### 2.3.2 Drug loading efficiency and *in vitro* drug releasing study

The encapsulation efficiency (EE) was calculated by the amount of the unencapsulated IgG in the supernatant after centrifugation. The concentration of unencapsulated IgG was detected by Nanodrop 1000 at 280 nm and Pierce™ BCA Protein Assay Kit (Thermofisher Scientific), following the Eq. (1) [12,32]:

(1) $$\mathrm{EE}\left(\%\right)=\frac{\mathrm{total amount of}\;\mathrm{IgG}-\mathrm{unencapsulated}\;\mathrm{IgG}}{\mathrm{total amount of}\;\mathrm{IgG}}\times 100\%$$

The drug loading efficiency (LE) was calculated following Eq. (2) [32]:

(2) $$\mathrm{LE}\left(\%\right)=\frac{t\mathrm{otal amount of}\;\mathrm{IgG}-\mathrm{unencapsulated}\;\mathrm{IgG}}{\mathrm{total amount of PLGA}}\times 100\%$$

The lyophilized microparticles (0.8 mg) were dispersed in 100 μL PBS (pH = 7.4) and incubated at 37 °C. At determined time intervals, the supernatants were collected by centrifugation at 12,000 rpm for 10 min [33]. The concentrations of released antibody were tested by Nanodrop 1000 at 280 nm.

#### 2.3.3 Detection of IgG stability by gel electrophoresis

10 mg lyophilized microparticles were dispersed in 600 μL PBS (pH = 7.4) and incubated at 37 °C. At determined time intervals, the supernatants were collected by centrifugation at 12,000 rpm for 10 min. The collected samples were added into an Ultra 30 kDa MWCO device (Amicon), and centrifuged for 20 min at 4000 ×*g* in a swinging bucket rotor to collect the purified and concentrated IgG solutions (20 μL).

20 μL of concentrated IgG solution were mixed with 10 μL of the non-reducing SDS (Thermofisher Scientific; J63615.AD), 4 μL of DTT (Thermofisher Scientific; R0861), and 6 μL of PBS. The samples were then incubated at 80 °C in a thermoblock for 5 min. 40 μL samples from different groups were loaded into individual wells of the Bis-Tris Plus gel (4–12 %) (Thermofisher Scientific; NW0412). Electrophoresis was carried out at 150 V for 45 min. The gel was then incubated into a suitable plastic container containing Coomassie blue solution (Lubio Science; LU001000) for 2 h, and destained in distilled water for 4 h prior to photography.

### 2.4 Polymeric film preparation

Polymer films were prepared using varying percentages of PLA, PCL, and PEG by solvent-casting procedure. The DCM was used as solvent. The appropriate ratios of constituents were dissolved in 10 mL of DCM (Table 2). The formulations were slowly mixed at 20 rpm to homogenise for 1 h and later cast in an 8-cm diameter glass petri dish. The petri dish was then placed in a fume cupboard at room temperature for 24 h for DCM evaporation.

**Table 2:** The overall concentration and composition of the polymeric films.

Formulation	Overall concentration (W/V%)	Composition (W/W%)		
		PLA	PCL	PEG
100 PLA_1	6 %	100	–	–
100 PCL_1	6 %	–	100	–
50 PLA:50 PCL_1	6 %	50	50	–
50 PLA:50 PEG_1	6 %	50	–	50
50 PCL:50 PEG_1	6 %	–	50	50
90 PCL:10 PEG_2	3 %	–	90	10
80 PCL:20 PEG_2	3 %	–	80	20
70 PCL:30 PEG_2	3 %	–	70	30
60 PCL:40 PEG_2	3 %	–	60	40
50 PCL:50 PEG_2	3 %	–	50	50

### 2.5 Preparing polymeric implant loaded with PLGA microspheres

The reservoir-type implants were fabricated by a heat-sealing process. The polymeric films were put under UV light for 1 h each side, and then cut into multiple small films. The reservoir-type implants were assembled by loading 0.8 mg of lyophilized PLGA microspheres between two polymeric films and heat-sealing the four edges. The final implants were approximately 3 mm × 3 mm in dimension. For the implants named F6-F10 and F21-F25, a PCL granule (0.3 mg) was melted and coated onto one side of the implant's surface. For the implants named F11-F15 and F26-F30, the PLGA granule was dissolved in DMSO (0.1 % *W*/*V*) and coated onto one side of the implant's surface.

### 2.6 Characteristics of the polymeric films

#### 2.6.1 Scanning electron microscopy

The scanning electron microscopy (SEM) analysis was taken using a Quanta 200 Emission Electron Microscope (FEI Company, USA). After coating with a 10 nm gold layer, samples were placed on an aluminum tube and the SEM images were taken at an operating voltage of 10.0 kV at different magnifications.

#### 2.6.2 Porosity analysis

In order to show the pore characteristics of the polymeric films, the pore size and pore density were measured by ImageJ version 1.53 and the pore size distribution was analyzed by SciDAVis 2.7.1.

The porosity of the polymeric films was analyzed as previously described [23]. The measuring cylinder containing 10 mL ethanol was weighed as M1 and the film was weighed as Ms. The film was immersed in the measuring cylinder containing ethanol and sonicated for 40 min to ensure the ethanol had penetrated into the pores of the film. After sonication, the ethanol volume in the cylinder was readjusted to 10 mL and the refilled cylinder was weighted as M2. The film saturated with the ethanol was then removed from the cylinder, and the measuring cylinder was weighed again as M3. The porosity (%) was calculated using the following Eq. (3):

(3) $$\mathrm{porosity}\left(\%\right)=\left[\frac{M2-M3-\mathrm{Ms}}{M1-M3}\right]\times 100$$

#### 2.6.3 Thickness of the polymeric films

The thickness of each film was measured by a digital calliper. Sixteen film squares (1 cm × 1 cm) were analyzed per film cast. To ensure that there was no gap present between the films, the polymeric film squares were clamped together securely using measuring bolts. The average thickness values were then calculated [34].

### 2.7 *In vitro* release studies and model fitting of drug release profiles

The implants prepared by polymeric films assembly were submerged in 100 μL PBS and placed in the incubator at 37 °C. At each predetermined time, the incubation buffer was collected and replaced by 100 μL of fresh PBS solution. The collected media were used for antibody measurements, and the concentrations of IgG released in the PBS media were measured using Nanodrop.

The *in vitro* drug release data were fitted to first order kinetics model (log cumulative of % drug remaining *vs.* time) and Higuchi model (cumulative % drug release *vs.* square root of time) [35]. The slope of the plot provided the first order rate constant and the release constant of Higuchi. The fit coefficient (R^2^) was employed for comparing the fitting accuracy of each model.

### 2.8 Degradation of the polymeric films

The polymeric films were placed in distilled water at 37 °C. At the defined time points, the films were thoroughly dried and the remaining weights of the films were measured. The films were then placed in distilled water at 37 °C. The weight loss ratio was calculated as the following Eq. (4):

(4) $$\mathrm{weight loss ratio}=\frac{\mathrm{Wt}}{W0}\times 100\%$$

where *W*_0_ and *W*_*t*_ are the initial weight and the weight determined at the time *t*, respectively.

### 2.9 Cell culture

Human trabecular meshwork (TM) cells were cultured from the trabecular meshwork tissues and human conjunctival fibroblasts (FF) were cultured from the conjunctival tissues of glaucoma patients after informed consent. All experiments were carried out following the rules of the Declaration of Helsinki and approved by the West of Scotland Research Ethics Committee (REC 19/WS/0146). The cells were cultured in the incubator at 37 °C with 5 % CO_2_ and 95 % humidity. The cells were then routinely passaged in T75 flasks using complete culture media consisting of DMEM, 10 % fetal calf serum, and 100 U/mL penicillin/0.1 mg/mL streptomycin.

### 2.10 Cell viability assay

Human FF and TM cells were plated in 96-well plates at a density of 6 × 10^3^ cells per well. The cells were treated with 50 μL complete media and 50 μL media containing the drug solution collected from each implant on days 1, 3, 5, 7, 14, 21 and 30. The next day, the cell media in all wells were replaced by 100 μL of fresh culture media per well, followed by the addition of 20 μL of CellTiter 96® AQueous One Solution Reagent (Southampton, UK). The plate was wrapped in foil and incubated for 2 h at 37 °C with 5 % CO_2_ and 95 % humidity. The plate was read using a plate reader, PHERAstar FS (BMG LABTECH), set at 490 nm absorbance, and the results were normalised against untreated cells. All experiments were performed as independent triplicates.

### 2.11 Statistical analysis

The model fittings were performed by Microsoft excel (version 16.78). Statistical data were analyzed as the mean ± standard error (SEM). Multiple comparisons were performed using one-way ANOVA using Prism 8. The values were considered significant at: *, *P* < 0.05; **, *P* < 0.01; ***, *P* < 0.001; ****, *P* < 0.0001; ns, not significant. All the experiments were performed as independent triplicates.

## 3 Results and discussions

### 3.1 Microparticle characterization

Water-soluble drugs are often encapsulated in PLGA microparticles using W_1_/O/W_2_ double emulsification, followed by lyophilization to enhance the stability of the microparticles. However, the double emulsion process can denature the IgG structure by approximately 27 %, and the lyophilization process can lead to structural changes of about 14.7 % [36]. The stability of the mAb can be optimized by adding various cryoprotectants. It has been reported that the recovery rate of 3D8 scFv in the primary emulsion added with 10 %*W*/*V* trehalose can reach 94.1 % [37]. Besides, Sousa et al. found that the addition of 10 %W/V trehalose not only maintained the secondary and tertiary structure and bioactivity of bevacizumab, but also retained PLGA microparticle stability and reduced microparticle aggregation after lyophilization [31]. Hence, we chose 10 %W/V trehalose as a cryoprotectant during the double emulsion process in this study.

**Fig. 1:**
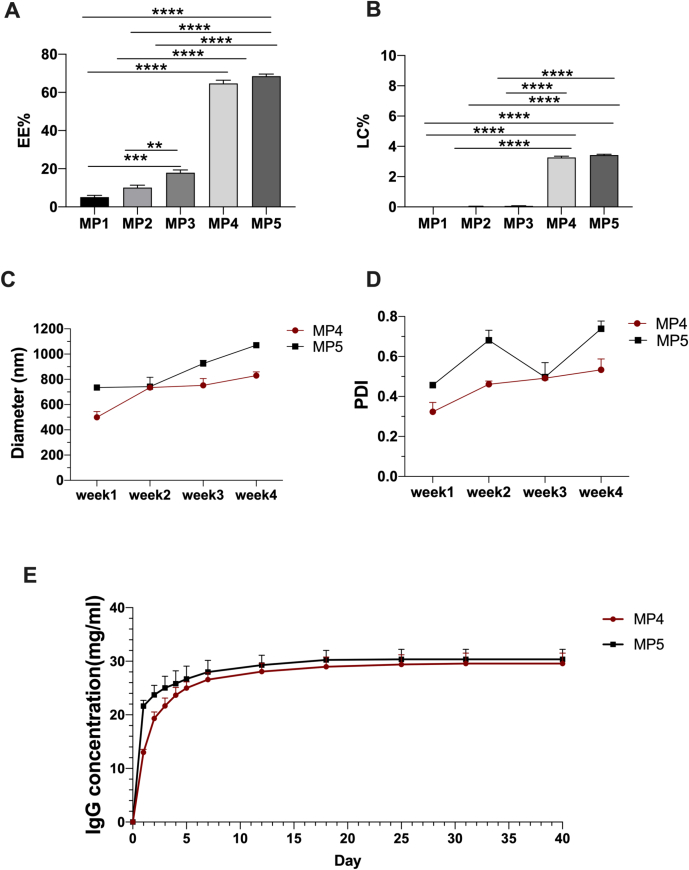
Effects of preparation parameters on the biophysical properties, drug loading efficacy and drug releasing effect of the PLGA microspheres. (A) Effect of different parameters on the drug encapsulation efficacy (EE). (B) Effect of different parameters on the drug loading capacity (LC). (C) Particle diameters of the PLGA microspheres with different PVA concentrations at weeks 1, 2, 3 and 4. (D) PDI of the PLGA microspheres with different PVA concentrations at weeks 1, 2, 3 and 4. (E) *In vitro* cumulative release rate of the PLGA microspheres with different PVA concentrations. **, *P* < 0.01; ***, *P* < 0.001; ****, *P* < 0.0001.

As a highly hydrophilic biologic, IgG is more prone to efflux from the organic/aqueous interphase to the external water phase before solidification of the microparticles. To improve the drug encapsulation efficiency, we optimized several formulation parameters to decrease the diffusion of the IgG from the organic droplets. In this study, EE% increased from 5.1 ± 0.9 % to 17.9 ± 1.5 %, and loading capacity% (LC%) increased from 0.017 ± 0.003 % to 0.070 ± 0.006 % by decreasing the PVA concentration from 2 % (MP1) to 0.5 % (MP3). Moreover, the addition of 5 %*W*/*V* NaCl significantly increased the EE% and LC% to 64.6 ± 1.7 % and 3.2 ± 0.1 %, respectively, with 1 % PVA in the external water phase (MP4). The EE% and LC% further increased to 68.5 ± 1.1 % and 3.4 ± 0.1 %, respectively, with 0.5 % PVA concentration and 5 %W/V NaCl (MP5) (Fig. 1A and B). No absorbance peak at 280 nm was detected in the plain PLGA microsphere group, hence the interference from impurities could be excluded (Table S2). Besides, the increase in EE% and LC% values by the addition of NaCl was also confirmed by the BCA assay (Fig. S1). This phenomenon might be explained by the osmotic effect of NaCl, which can depress the hydrophilic drug solubility in the external phase [38]. In addition, the NaCl concentration in the external aqueous phase can also lead to the formation of faster, solidifying, crust-like structure on the surface of PLGA microparticles, which can form as a barrier and impede drug leakage, thereby enhancing the drug loading and yield rate [39].

The microparticle size and polydispersity index (PDI) are crucial factors that determine the stability of the PLGA microspheres [40]. We thus monitored the microparticle size and PDI of MP4 and MP5 every week for one month. After preparation, the microspheres were collected and showed the average size of 499.7 ± 45.4 μm for MP4 and 735.3 ± 19.7 μm for MP5. The average size value slightly increased when the microparticles were stored in water at 4 °C for 28 days, and the mean diameters of MP4 and MP5 increased to 830.0 ± 28.9 μm and 1070.7 ± 19.1 μm, respectively. This increase was more visible in MP5 and was attributed to the higher PVA concentration in the external phase, which promotes the formation of more stable emulsion droplets [41] (Fig. 1C).

PDI is used to assess the uniformity of the particle size distribution. In general, the sample is considered to be highly monodisperse when the PDI is below 0.5, and a PDI above 0.7 indicates that the particles have a broad particle size distribution [42]. Upon preparation, both MP4 and MP5 showed uniform appearances, as indicated by a PDI <0.5. The PDI increased to 0.5 ± 0.1 after storing MP4 for 4 weeks in water, while the PDI of MP5 was increased to 0.8 ± 0.1, indicating that there were likely aggregation and fusion of MP5 [36]. These findings are consistent with the experimental results reported by Pandit et al. [43,44]. This observation may also be attributed to the stabilizing effect of PVA, where a higher concentration of PVA correlates with a lower PDI value (Fig. 1D).

Fig. 1F illustrates the biphasic drug release profiles from MP4 and MP5, characterized by burst release followed by a decreased release rate of IgG. Here, we observed that as the PVA concentration increased from 0.5 % *W*/*V* to 1 % W/V in the continuous phase, the burst release of IgG decreased from 78.15 % to 65.39 %. This phenomenon can be attributed to the higher PVA concentrations yielding a more stable emulsion, which inhibits the mass transfer of IgG to the surroundings and thus reduces the drug burst release [41]. We then investigated the structural integrity of the IgG released from MP4 using sodium dodecyl sulfate–polyacrylamide gel electrophoresis (SDS-PAGE) [45]. Our results revealed two distinct bands corresponding to the heavy and light chains of IgG in each sample, confirming the preservation of IgG stability during microparticle fabrication (Fig. S2). As MP4 achieved a more controlled and prolonged release compared to MP5, MP4 was therefore used in the following experiments.

### 3.2 Characterization of the polymeric films composed of plain PLA (100 PLA), plain PCL (100 PCL), 50 PLA: 50 PCL, 50 PLA: 50 PEG, 50 PCL: 50 PEG

**Fig. 2:**
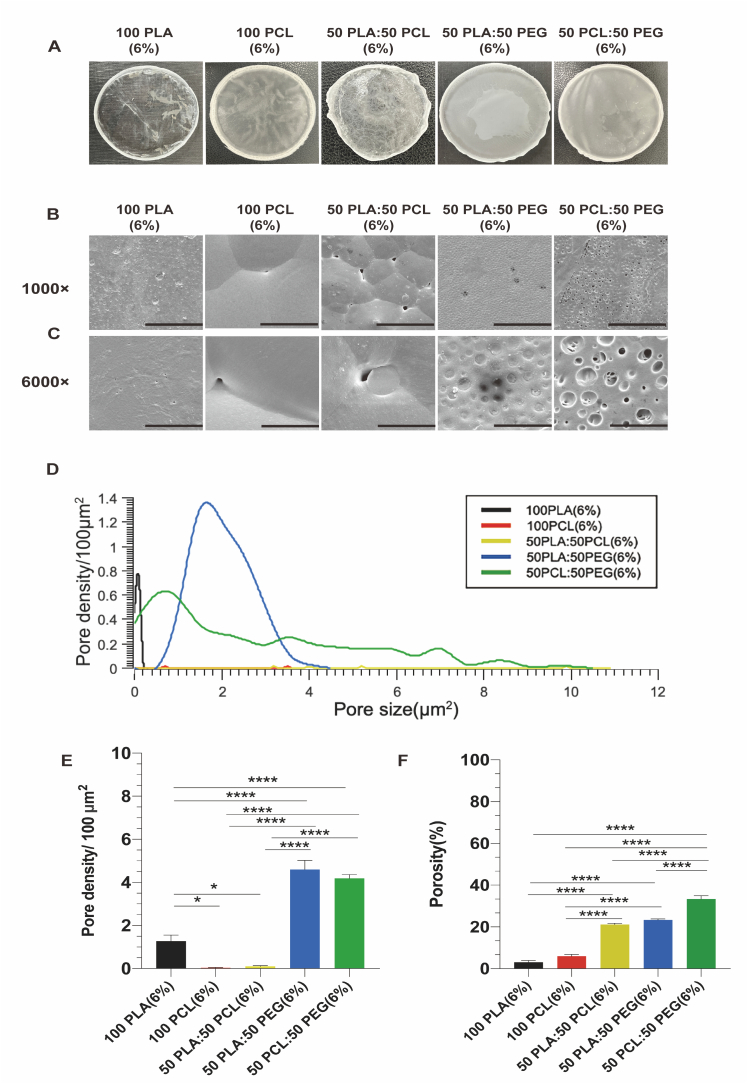
Characteristics of plain PLA (6 %), plain PCL (6 %), 50 PLA: 50 PCL (6 %), 50 PLA: 50 PEG (6 %), 50 PCL: 50 PEG (6 %). (A) Images of the polymeric films with different compositions prepared by a solvent-casting method. (B–C) SEM images of polymeric films prepared by a solvent-casting method with magnifications of (B) 1000×, scale bar, 50 μm. (C) 6000×, scale bar, 10 μm. (D) Pore size distribution of polymeric films. (E) Pore density of polymeric films. (F) Porosity of polymeric films. Results represent mean ± SEM, *N* = 3. *, *P* < 0.05; ****, *P* < 0.0001.

Polymeric materials are extensively used in the development of drug delivery formulations owing to their numerous advantages, including biocompatibility, biodegradability, flexibility, and ease of processing and shaping [46]. Solvent-casting method is one of the most common approaches for the preparation of polymeric films. Apart from the transparent and rigid nature of the plain PLA film, it was evident that the remaining four films exhibited an opaque and oily appearance (Fig. 2A). In contrast to the film derived from PCL, the film produced from PLA exhibited greater rigidity. This disparity arises from the fact that PLA possesses a higher glass transition temperature of 60 °C, resulting in a high modulus at room temperature. Conversely, PCL displays a lower glass transition temperature of −60 °C, rendering it less resistant to tensile forces and softer under room temperature conditions [47].

Scanning electron microscopy was used to analyze the surface topography in different films. From the SEM analysis, the surface of the pure PLA fabricated by solvent-casting method had small surface pits with irregular shapes, which might be a result of solvent evaporation during the drying cycle [48]. The pure PCL film exhibited smooth surface with several pores, and these pores were varied in size and were identified as gaps between the surface PCL spherulites [49]. Compared to the pure PLA or pure PCL films, the films composed of PLA, PCL and PEG blends were more suitable to be used as the controlled-release membrane due to their more porous structure. The pores formed in the 50 PLA: 50 PCL film are due to the macrophase separation that occurs between the two immiscible polymers [47]. In blends of hydrophobic (PLA or PCL) and hydrophilic (PEG) polymers, the lower surface energy component (PLA or PCL) is more prone to dominate the surface, while PEG tends to disperse spherically within the PLA or PCL phase to minimize interfacial energy due to its high hydrophilicity and mobility [27,50,51]. These behaviors might help to explain the distinct spherical pore formation observed in the membranes that blend PEG with PLA and PCL (Fig. 2B–C).

Subsequently, an analysis of pore size distribution and pore density among different film surfaces was carried out (Fig. 2D–E). The 100 PLA film and 100 PCL film showcased a narrow pore size distribution, with the pore sizes ranging from 0 to 0.2 μm^2^ and 0.7 to 3.6 μm^2^, respectively. Conversely, the film consisting of a 50:50 ratio of PLA to PCL exhibited the broadest pore size distribution, ranging from 3.2 to 11.9 μm^2^. The film comprising of a 50:50 blend of PLA and PEG presented a pore size distribution ranging from 0.8 to 4.1 μm^2^ with a pore density of 4.6 ± 0.4/100 μm^2^. The film composed of 50 % PCL and 50 % PEG displayed a wide pore size distribution ranging from 0.1 to 10.3 μm^2^ with a pore density of 4.2 ± 0.2 pores/100 μm^2^. These observations collectively suggested that the composition of polymers significantly influenced the structural characteristics of the films.

We next analyzed porosity as it is a critical factor influencing drug release [27]. It was evident that both the 100 PLA film and 100 PCL film exhibited dense structures, with porosities measured at 3.1 ± 0.8 % and 6.0 ± 0.8 %, respectively. Conversely, the films consisting of polymer blends, such as 50 PLA:50 PCL, 50 PLA:50 PEG, and 50 PCL:50 PEG, demonstrated porous structures and had porosity levels of 21.2 ± 0.5 %, 23.3 ± 0.5 %, and 33.3 ± 1.7 %, respectively (Fig. 2F). However, it should be noted that during porosity measurement, the dissolution of PEG by ethanol might affect the results of porosity analysis for the polymeric films containing PEG.

These results suggested that the structure of polymeric films can be tailored by changing the polymer mixture, and that polymer blends normally produced more porous films compared to the films made of a single polymer.

### 3.3 Preparation of the polymeric implants

**Fig. 3:**
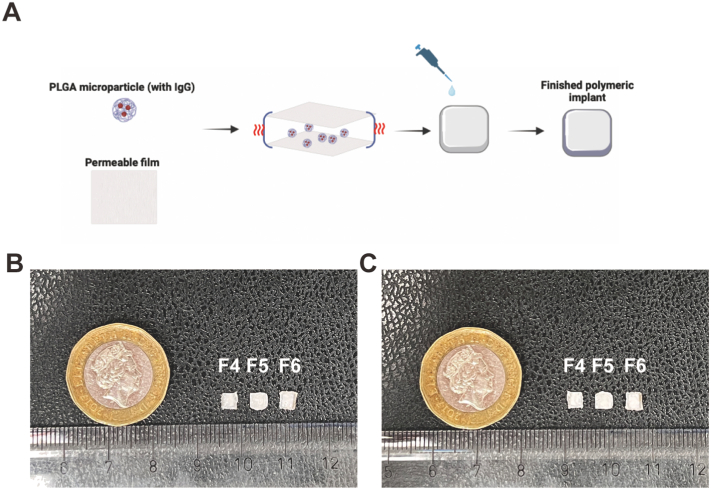
Finished polymeric implants. (A) Schematic showing the fabricating process of the reservoir-type polymeric implants. (B) Morphology of the polymeric implant (Top surface). (C) Morphology of the polymeric implant (Bottom surface).

To develop the IgG sustained-release system, the films mentioned in the previous section were assembled by a heat-sealing process. 0.8 mg of lyophilized PLGA microparticles with encapsulated IgG was sandwiched in a polymer membrane pocket, followed by coating of the implant with 1 layer of molten PCL or 1 layer of 0.1 %*W*/*V* PLGA solution (Fig. 3A). It is important to note that the molten PCL encapsulating the microporous film does not possess its own porosity, thereby restricting drug permeation solely through the sustained-release membrane. The implants had similar dimensions to the intracameral implant previously reported by Kim *et al.* [52] (Fig. 3B–C).

### 3.4 *In vitro* drug release profiles of the polymeric implants

**Fig. 4:**
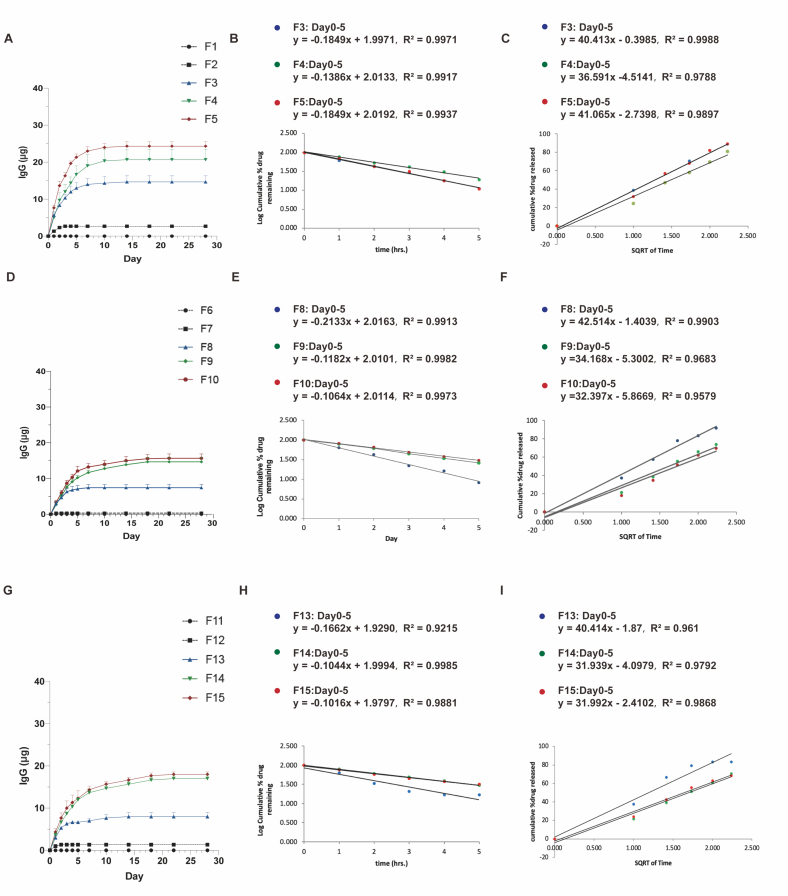
IgG *in vitro* release profiles for formulations F1–F15. (A) *In vitro* release profiles of IgG from formulations F1–F5. (B) First order release kinetics of IgG from formulations F1–F5. Model fitting equations and corresponding R^2^ values are presented. (C) Higuchi release kinetics of IgG from formulations F1–F5. Model fitting equations and corresponding R^2^ values are presented. (D) *In vitro* release profiles of IgG from formulations F6–F10. (E) First order release kinetics of IgG from formulations F6–F10. Model fitting equations and corresponding R^2^ values are presented. (F) Higuchi release kinetics of IgG from formulations F6–F10. Model fitting equations and corresponding R^2^ values are presented. (G) *In vitro* release profiles of IgG from formulations F11–F15. (H) First order release kinetics of IgG from formulations F11–F15. Model fitting equations and corresponding R^2^ values are presented. (I) Higuchi release kinetics of IgG from formulations F11–F15. Model fitting equations and corresponding R^2^ values are presented.

We next evaluated the release kinetics of each implant (Fig. 4). In theory, the permeation of the film is proportional to the porosity. Due to less porosity, the implants composed of 100 PLA film (F1, F6, F11) and 100 PCL film (F2, F7, F12) showed a negligible drug release profile. For the implants composed of 50 PLA:50 PCL (F3, F8, F13), 50 PLA:50 PEG (F4, F9, F14) and 50 PCL:50 PEG (F5, F10, F15), all formulations demonstrated a burst release initially, followed by a gradual and sustained release over time. Notably, 50 PCL:50 PEG implants with PLGA coating barrier (F15) showed the longest drug release profile with the least drug remaining in the implant, where about 79.44 % were released by the end of 7 days and the implant then showed a continuous IgG release pattern for approximately one week. 50 PLA:50 PEG implant with PLGA solution coating (F14) also showed a long drug release profile lasting up to 18 days. However, compared to F15, F14 had more drug retention in the implant (16.3 ± 0.8 μg released from F14 compared to 18.0 ± 0.5 μg released from F15 over 4 weeks).

In order to further characterize the drug release pattern of the drug delivery formulations, the first-order release model and Higuchi model were next applied to evaluate the drug release. The first-order release model is an equation to fit the concentrated-dependent release rate [35,53]. On the other hand, the Higuchi model is utilized to describe the cumulative amount of drug release from a film-controlled drug delivery system, where the release is proportional to the square root of time [54,55]. Our results showed that, apart from F3 and F13 that were more fitted to the Higuchi model, all the other formulations fitted well with the first-order kinetic model, indicating that the mechanisms of IgG released from the polymeric film are concentration dependent.

In this study, we tested the polymeric implants without perimeter setting, and with molten PCL layer or PLGA solution layer. Our results showed that the drug releasing profiles changed by modifying the perimeter settings during the implant fabrication process. The implants coated with molten PCL layer demonstrated minimal drug release, with only 0.3 ± 0.3 μg (F7), 7.4 ± 0.9 μg (F8), 15.7 ± 1.2 μg (F9), and 14.7 ± 1.3 μg (F10) of IgG released over 4 weeks, possibly due to their lower membrane permeability. Conversely, the implants without any coating exhibited the most sustained IgG release, with 2.6 ± 0.3 μg (F2), 14.6 ± 1.6 μg (F3), 20.6 ± 2.8 μg (F4), and 24.3 ± 1.2 μg (F5) of IgG released over 30 days. For the implants coated with PLGA barrier layer, there were about 1.3 ± 0.3 μg (F12), 8.0 ± 1.0 μg (F13), 16.3 ± 0.8 μg (F14), and 18.0 ± 0.5 μg (F15) of IgG released over 4 weeks (Fig. S3 and Table 3).

**Table 3:** The characteristics of different polymeric implants prepared using different parameters. Results represent mean ± SEM, *N* = 3.

	Composed film		Implant				
	Composition	Thickness (μm)	Weight (mg)	Modification	MP loading (mg)	Theoretical drug loading (μg)	Actual drug releasing (μg)
F1	100 PLA_1	43.7	3.6 ± 0.1	NA	0.8	28.7 ± 2.4	0.0
F2	100 PCL_1	56.2	3.6 ± 0.1	NA	0.8	28.7 ± 2.4	2.6 ± 0.3
F3	50 PLA:50 PCL_1	68.7	3.6 ± 0.1	NA	0.8	28.7 ± 2.4	14.6 ± 1.6
F4	50 PLA:50 PEG_1	75.0	3.5 ± 0.1	NA	0.8	28.7 ± 2.4	20.6 ± 2.8
F5	50 PCL:50 PEG_1	81.2	3.6 ± 0.1	NA	0.8	28.7 ± 2.4	24.3 ± 1.2
F6	100 PLA_1	43.7	4.3 ± 0.2	Molten PCL	0.8	28.7 ± 2.4	0.0
F7	100 PCL_1	56.2	4.6 ± 0.1	Molten PCL	0.8	28.7 ± 2.4	0.3 ± 0.3
F8	50 PLA:50 PCL_1	68.7	4.5 ± 0.1	Molten PCL	0.8	28.7 ± 2.4	7.4 ± 0.9
F9	50 PLA:50 PEG_1	75.0	4.3 ± 0.1	Molten PCL	0.8	28.7 ± 2.4	15.7 ± 1.2
F10	50 PCL:50 PEG_1	81.2	4.6 ± 0.1	Molten PCL	0.8	28.7 ± 2.4	14.7 ± 1.3
F11	100 PLA_1	43.7	3.6 ± 0.1	PLGA solution	0.8	28.7 ± 2.4	0.0
F12	100 PCL_1	56.2	3.6	PLGA solution	0.8	28.7 ± 2.4	1.3 ± 0.3
F13	50 PLA:50 PCL_1	68.7	3.7	PLGA solution	0.8	28.7 ± 2.4	8.0 ± 1.0
F14	50 PLA:50 PEG_1	75.0	3.7 ± 0.1	PLGA solution	0.8	28.7 ± 2.4	16.3 ± 0.8
F15	50 PCL:50 PEG_1	81.2	3.6 ± 0.1	PLGA solution	0.8	28.7 ± 2.4	18.0 ± 0.5
F16	90 PCL:10 PEG_2	37.5	3.6 ± 0.1	NA	0.8	28.7 ± 2.4	4.0 ± 0.0
F17	80 PCL:20 PEG_2	37.5	3.7 ± 0.1	NA	0.8	28.7 ± 2.4	14.6 ± 0.8
F18	70 PCL:30 PEG_2	37.5	3.7 ± 0.1	NA	0.8	28.7 ± 2.4	25.3 ± 0.6
F19	60 PCL:40 PEG_2	37.5	3.6 ± 0.1	NA	0.8	28.7 ± 2.4	26.3 ± 0.3
F20	50 PCL:50 PEG_2	37.5	3.6 ± 0.1	NA	0.8	28.7 ± 2.4	27.2 ± 0.8
F21	90 PCL:10 PEG_2	37.5	4.5 ± 0.1	Molten PCL	0.8	28.7 ± 2.4	2.3 ± 0.7
F22	80 PCL:20 PEG_2	37.5	4.7 ± 0.1	Molten PCL	0.8	28.7 ± 2.4	8.7 ± 2.3
F23	70 PCL:30 PEG_2	37.5	4.5 ± 0.1	Molten PCL	0.8	28.7 ± 2.4	14.0 ± 0.7
F24	60 PCL:40 PEG_2	37.5	4.4 ± 0.1	Molten PCL	0.8	28.7 ± 2.4	16.3 ± 0.6
F25	50 PCL:50 PEG_2	37.5	4.6 ± 0.1	Molten PCL	0.8	28.7 ± 2.4	17.9 ± 0.9
F26	90 PCL:10 PEG_2	37.5	3.6	PLGA solution	0.8	28.7 ± 2.4	4.0 ± 0.5
F27	80 PCL:20 PEG_2	37.5	3.6	PLGA solution	0.8	28.7 ± 2.4	10.6 ± 0.6
F28	70 PCL:30 PEG_2	37.5	3.6 ± 0.1	PLGA solution	0.8	28.7 ± 2.4	18.0 ± 1.1
F29	60 PCL:40 PEG_2	37.5	3.6 ± 0.1	PLGA solution	0.8	28.7 ± 2.4	24.0 ± 1.0
F30	50 PCL:50 PEG_2	37.5	3.6 ± 0.1	PLGA solution	0.8	28.7 ± 2.4	26.0 ± 0.5

It should be noted that 100 PLA (6 %) polymeric film is not suitable for use as a drug-controlled release material as there is almost no drug release from the implants composed of 100 PLA (6 %) polymeric film. On the other hand, the implants containing a ratio of 50 PCL: 50 PEG (6 %), with PLGA coating exhibited the longest continuous release time, which lasted almost 18 days and with approximately 62.7 % of the IgG released.

### 3.5 Changes in the polymeric films after 30-day drug release experiment

**Fig. 5:**
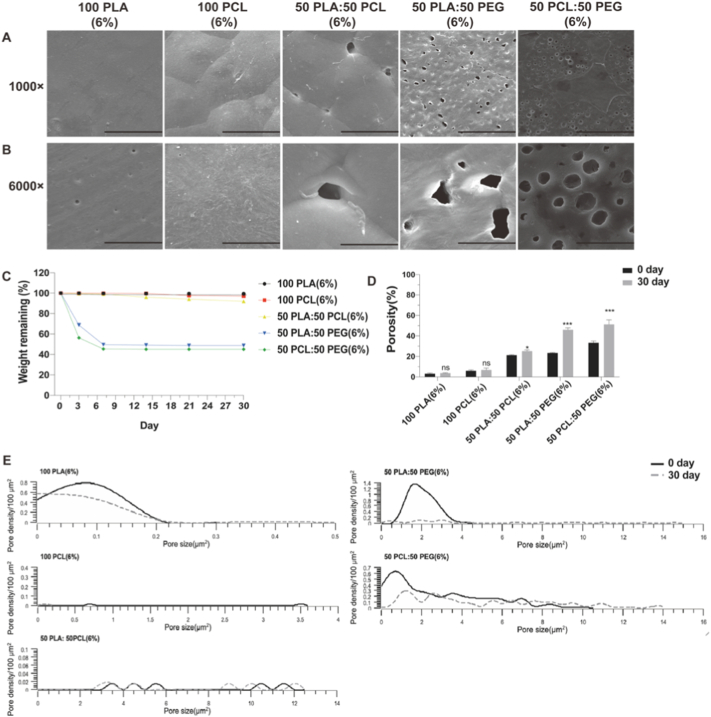
Weight changes, pore size distribution, pore density and porosity of the polymeric film after the drug release experiment. (A–B) SEM of the films prepared by a solvent-casting method after the drug release experiment with magnifications of (A) 1000×, scale bar, 50 μm. (B) 6000×, scale bar, 10 μm. (C) Weight loss of the polymeric films prepared by a solvent-casting method from day 0 to day 30. Results represent mean ± SEM, *N* = 3. (D) Porosity of the films measured at the beginning (day 0) and at the end (day 30) of the drug release experiment. Results represent mean ± SEM, N = 3. *, *P* < 0.05; ***, *P* < 0.001; ns, not significant. (E) Pore size distribution of films measured at the beginning (day 0) and at the end (day 30) of the drug release experiment. N = 3.

Next, we conducted a detailed assessment of the morphological changes in the polymeric films after the drug release experiment. The SEM results showed that the surface pore tomography of 100 PLA (6 %), 100 PCL (6 %), and 50 PLA: 50PCL (6 %) films remained relatively unaltered after the 30-day drug release assay. In contrast, the pore sizes of the 50 PLA: 50 PEG (6 %) film and 50 PCL: 50 PEG (6 %) film exhibited noticeable enlargement, potentially attributed to the leaching of PEG from the films (Fig. 5A–B).

Moreover, we further assessed the degradation profile of the five polymeric films over 30 days (Fig. 5C). However, due to the hydrophobicity properties of PLA and PCL, the degradation rates in the PLA and PCL films were very slow in the first two weeks at about 0.5 ± 0.3 % and 0.2 ± 0.1 %, respectively, which were negligible. From day 14 to day 30, the degradation rate of the PCL film increased with time and showed a change in the weight loss to 4.0 ± 1.0 % at the end of 30 days, indicating that the PCL hydrolysis rate had increased. Blending PCL with PLA had little impact on the weight loss initially [56], and 50 PLA: 50 PCL film lost 8.0 ± 0.7 % of its weight in 30 days. Hence, we concluded that due to the hydrophobic properties of PLA and PCL, the pure PLA and PCL films and 50 PLA:50 PCL film exhibited a slow degradation rate, which was mainly attributed to the polymer chain scission and the dissolution of the oligomers [57]. However, the films where we blended PLA or PCL with PEG underwent a rapid rate of degradation. The major weight loss of 50 PLA:50 PEG and 50 PCL:50 PEG films occurred in the first week to 50.5 ± 0.7 % and 54.3 ± 1.3 %, respectively, and reached a plateau thereafter; and there were still 48.8 ± 0.9 % and 45.1 ± 1.2 % of the original weight remaining at the end of the 30 days.

After immersion in the PBS for 30 days, the 50 PLA:50 PCL (6 %), 50 PLA:50 PEG (6 %), and 50 PCL:50 PEG (6 %) films showed an increase in porosity by 4.1 %, 22.6 %, and 17.9 %, respectively, which was particularly marked for the 50 PLA:50 PEG (6 %) and 50 PCL:50 PEG (6 %) films. Conversely, the porosity changes in the 100 PLA (6 %) and 100 PCL (6 %) films were not statistically significant (Fig. 5D).

The surface pore characteristic was then quantified by performing pore size distribution analysis. It should be noted that, due to the leaching out of the PEG, the surface pore distribution of 50 PLA:50 PEG (6 %) and 50 PCL:50 PEG (6 %) after the drug release experiment exhibited significant enlargement compared to the original films. The pore sizes on the original 50 PLA:50 PEG (6 %) film had a peak value centered around 0.8 to 4.1 μm^2^, whereas the maximal size of the pores increased from 4.1 μm^2^ to 14.7 μm^2^ after the drug release experiment. The initial 50 PCL:50 PEG (6 %) film exhibited numerous small-sized pores that ranged from 0.1 to 10.3 μm^2^. After the drug release experiment, there was a slight increase in pore size distribution, ranging from 0.2 to 14.0 μm^2^ (Fig. 5E).

**Fig. 6:**
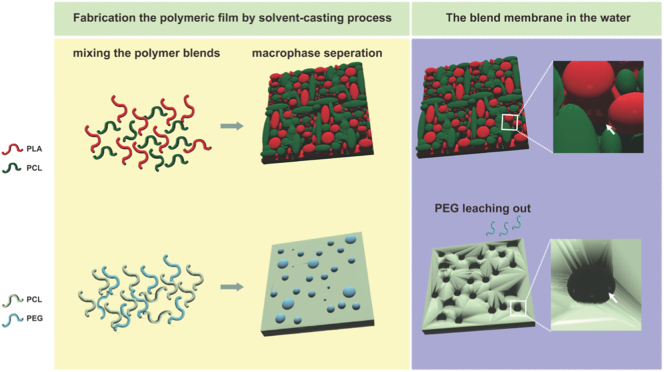
Macrophase separation and degradation behavior of polymeric blends. White arrows indicate the pore formation in the polymeric membrane.

The fabrication of microporous polymeric films based on the macrophase separation theory is frequently employed to modulate the rate of drug release in drug delivery systems. This theory refers to when immiscible polymers are mixed, each component can spontaneously separate into distinct macroscopic domains, forming a single polymeric material with interpenetrating structures [58]. However, the selection of different blend materials is crucial as it significantly impacts the associated physical properties, such as porosity, pore size and pore interconnectivity, and therefore influences the final product. In this study, we found that due to the intrinsic immiscibility of PLA, PCL and PEG, the blend of different polymers tended to separate into distinct phases rather than form a uniform mixture. The macrophase separation of the polymer blends and the poor adhesion after solvent evaporation between the two phases can contribute to the formation of voids or pores within the polymeric films [47,[57], [58], [59]]. Furthermore, the highly hydrophilic nature of PEG can result in its leaching from the polymeric matrix when incubated in water, thereby causing a more porous and loose structure. In contrast, blending two hydrophobic polymers together typically yields a more stable structure that is less prone to degradation (Fig. 6).

### 3.6 Further characterization of the polymeric films with different PCL:PEG ratios

As the 50 PCL:50 PEG (6 %) film showed a strong likelihood of pore formation and the implants containing this composition demonstrated excellent drug release efficacy over 30 days, we selected PCL and PEG as the primary retarding polymers to fabricate the implants with maximal IgG release. The polymeric films composed of varying PCL:PEG ratios, including 90:10 (3 % *W*/*V*), 80:20 (3 % W/V), 70:30 (3 % W/V), 60:40 (3 % W/V), and 50:50 (3 % W/V), were therefore further investigated in the following experiments.

**Fig. 7:**
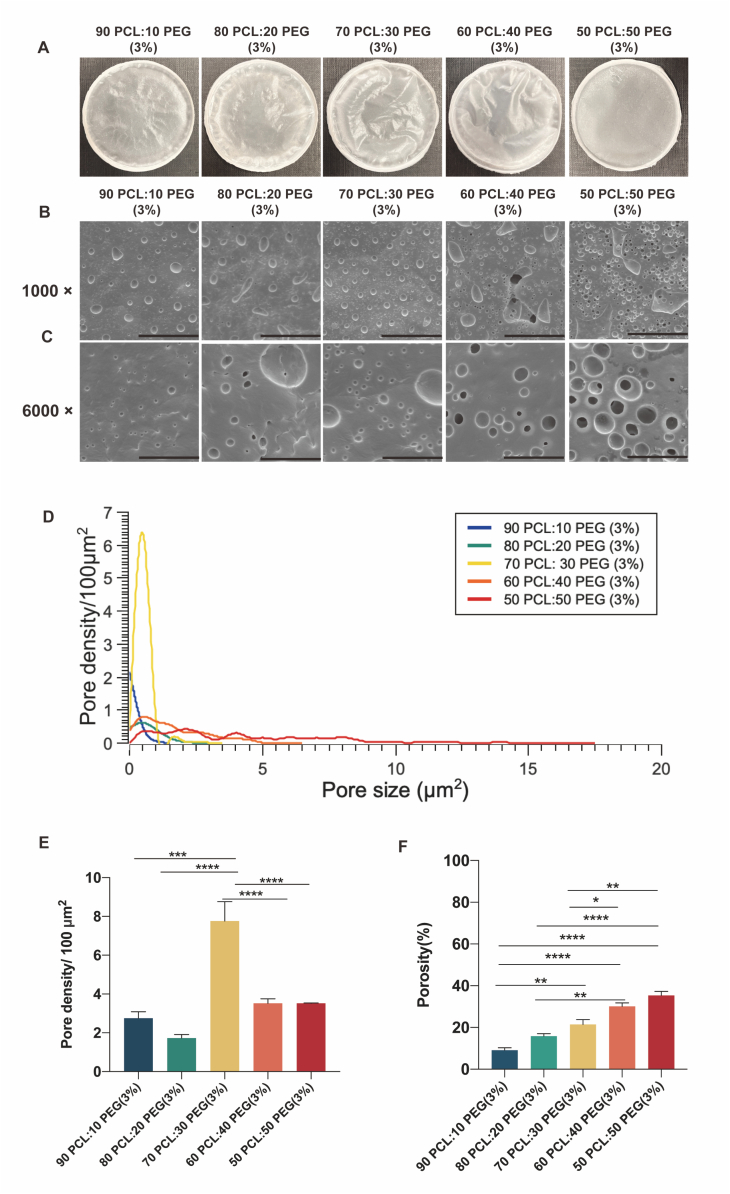
Characteristics of the polymeric films with different ratios of PCL/PEG. (A) Images of the polymeric films with different ratios of PCL/PEG. (B–C) SEM images of PCL/PEG polymeric films prepared by a solvent-casting method with magnifications of (B) 1000×, scale bar, 50 μm. (C) 6000×, scale bar, 10 μm. (D) Pore size distribution of PCL/PEG polymeric films prepared by a solvent-casting method. (E) Pore density of PCL/PEG polymeric films prepared by a solvent-casting method. (F) Porosity of PCL/PEG polymeric films prepared by a solvent-casting method. Results represent mean ± SEM, *N* = 3. *, *P* < 0.05; **, *P* < 0.01; ***, *P* < 0.001; ****, *P* < 0.0001.

The polymeric films with different PCL and PEG ratios were prepared using solvent-casting methods (Fig. 7A). The surface morphologies of the films were analyzed by SEM scanning. Consistent with previously published results, the degree of pore formation and porosity were dependent on the amount of PEG [27]. As the PEG concentration increased, the film surface showed more pores and exhibited a certain degree of surface deformation (Fig. 7B–C).

The pore size distribution and pore density of PCL films with different PEG ratios are shown in Fig. 7D and E. Among the compositions tested, the 90 PCL:10 PEG (3 %) film exhibited the narrowest pore size distribution, ranging from 0 to 1 μm^2^, with a pore density of 2.75 ± 0.33 pores/100 μm^2^. Following this, the 80 PCL:20 PEG (3 %) film displayed a slightly broader distribution (0 to 3 μm^2^) with a pore density of 1.7 ± 0.2 pores/100 μm^2^. Interestingly, the surface of the 70 PCL:30 PEG (3 %) film showcased the formation of numerous small pores (0 to 3 μm^2^), with the highest pore density observed at 7.8 ± 1.0 pores/100 μm^2^. As the PEG content increased further, such as in the case of the 60 PCL:40 PEG (3 %) and 50 PCL:50 PEG (3 %) films, a trend towards the merging of small pores into larger ones became evident. Consequently, the surfaces of the 60 PCL:40 PEG (3 %) and 50 PCL:50 PEG (3 %) films exhibited a wider range of pore sizes, spanning from 0 to 6.5 μm^2^ and 0 to 17.0 μm^2^, respectively, with a corresponding decrease in surface pore density to 3.5 ± 0.2 pores/100 μm^2^ and 3.5 pores/100 μm^2^, respectively.

Besides, the porosity analysis also confirmed that with increasing PEG concentration, there was a corresponding increase in the pore formation in the polymeric film. The porosities of 90 PCL:10 PEG (3 %), 80 PCL:20 PEG (3 %), 70 PCL:30 PEG (3 %), 60 PCL:40 PEG (3 %), and 50 PCL:50 PEG (3 %) films were 9.1 ± 1.2 %, 15.9 ± 1.1 %, 21.4 ± 2.4 %, 30.2 ± 1.7 % and 35.4 ± 1.9 %, respectively (Fig. 7F).

### 3.7 *In vitro* drug release study of the polymeric implants composed of PCL/PEG polymeric films

**Fig. 8:**
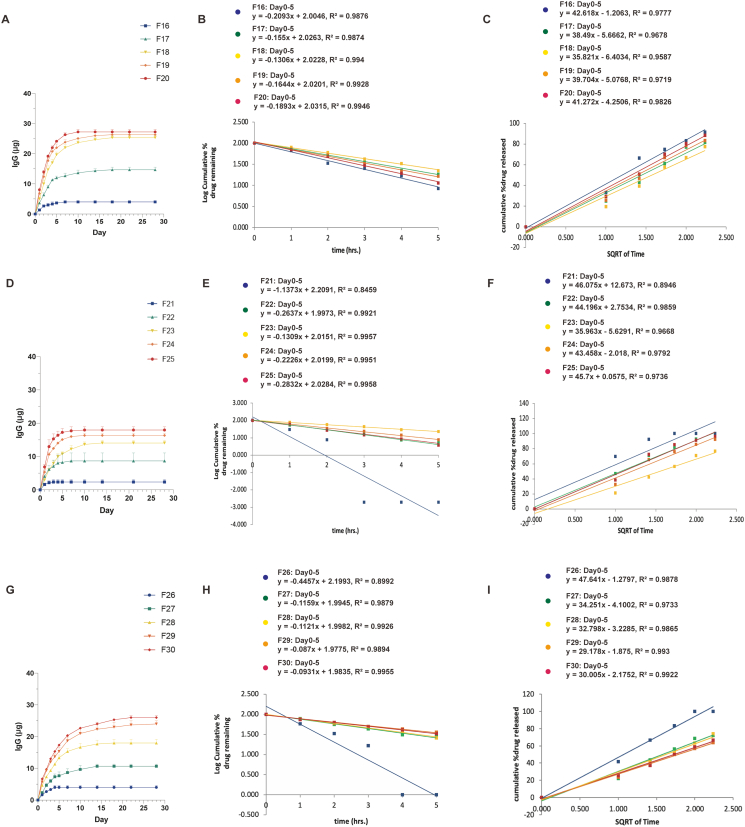
*In vitro* release profiles of IgG for the formulations F16–F30 (A) *In vitro* release profiles of IgG from the formulations F16–F20. (B) First order release kinetics of IgG from the formulations F16–F20. Model fitting equations and corresponding R^2^ values are presented. (C) Higuchi release kinetics of IgG from the formulations F16–F20. Model fitting equations and corresponding R^2^ values are presented. (D) *In vitro* release profiles of IgG from the formulations F21–F25. (E) First order release kinetics of IgG from the formulations F21–F25. Model fitting equations and corresponding R^2^ values are presented. (F) Higuchi release kinetics of IgG from the formulations F21–F25. Model fitting equations and corresponding R^2^ values are presented. (G) *In vitro* release profiles of IgG from the formulations F26–F30. (H) First order release kinetics of IgG from the formulations F26–F30. Model fitting equations and corresponding R^2^ values are presented. (I) Higuchi release kinetics of IgG from the formulations F26–F30. Model fitting equations and corresponding R^2^ values are presented.

Fig. 8 represents the drug release profiles in different polymeric implant formulations. Notably, F29 and F30 were the most effective continuous delivery systems, releasing sustained high levels of IgG over 4 weeks. Formulations lacking any coating layer (F16-F20) exhibited first-order kinetics. Among these, F16 and F20 comprised of the 50 PCL: 50 PEG (3 %) film and 90 PCL: 10 PEG (3 %) film, respectively, demonstrated the fastest elution of IgG and the cessation of IgG release by the end of day 7 and day 10, respectively. The high porosity of the 50 PCL: 50 PEG (3 %) film facilitated rapid passive diffusion of IgG from F16, whereas the limited porosity of the 90 PCL: 10 PEG (3 %) film restricted passive diffusion from F20. For the implants F17, F18, and F19, the released IgG reached 97.7 %, 97.3 %, and 98.8 %, respectively, by the end of day 14 (Fig. 8A–C).

Among the implants coated with a PCL molten layer (F21, F22, F23, F24, and F25), apart from the F21 which did not conform to either first-order release kinetics (R^2^ = 0.8459) or the Higuchi model (R^2^ = 0.8946), all the other implants displayed concentration-dependent release profiles, aligning more closely with first-order release kinetics. Notably, among all the polymeric formulations, F23 provided the best results for sustained drug release, maintaining continuous IgG release over a 14-day period (Fig. 8D–F).

The implants coated with a PLGA layer exhibited distinct release profiles: F26 and F29 conformed more closely to the Higuchi model, while F27, F28, and F30 fitted well with the first-order kinetic model. By the end of day 5, only 64.7 % and 66.7 % of IgG had been released from F29 and F30, respectively. However, by day 14, these figures had risen significantly to 93.1 % and 92.3 % for F29 and F30, respectively. Interestingly, F29 demonstrated higher drug release efficacy and sustained release for about 4 weeks (Fig. 8G–H).

Besides, it became evident that with increasing PEG concentration, there was a corresponding increase in the IgG release. Notably, the 90 PCL:10 PEG implant demonstrated minimal drug release, with only 4.0 μg (F16), 2.3 ± 0.7 μg (F21) and 4.0 ± 0.5 μg (F26) of IgG released over 4 weeks, possibly due to their lower membrane permeability. In contrast, the 50 PCL:50 PEG implant exhibited the highest sustained release, with nearly 27.2 ± 0.8 μg (F20) 17.9 ± 0.9 μg (F25), and 26.0 ± 0.5 μg (F30) of IgG released over 4 weeks (Fig. S4 and Table 3).

By modifying the membrane porosity and perimeter settings, our results showed that the implant containing a ratio of 60 % PCL to 40 % PEG (3 %) and coated with 0.1 % *W*/*V* PLGA exhibited the most effective sustained-release property. This implant formulation could sustained-release 83.6 % of IgG over 4 weeks.

### 3.8 Changes of the PCL/PEG polymeric films after 30-day drug release experiment

**Fig. 9:**
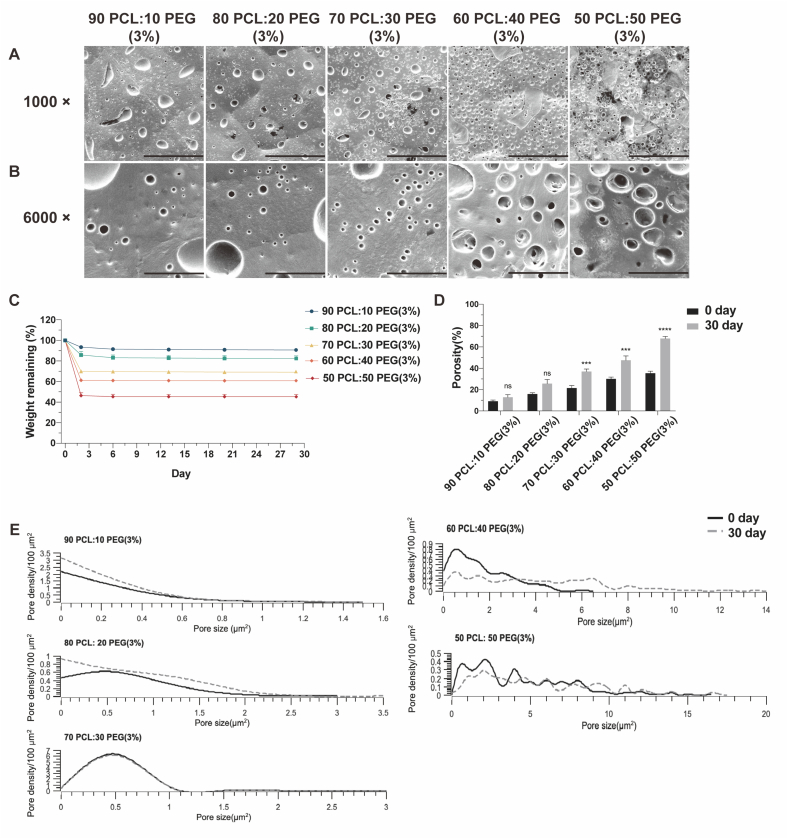
Weight changes, pore size distribution, pore density and porosity of the PCL/PEG polymeric films after the drug release experiment. (A-B) SEM of the PCL/PEG films prepared by a solvent-casting method after the drug release experiment with magnifications of (A) 1000×, scale bar, 50 μm. (B) 6000×, scale bar, 10 μm. (C) Weight loss of the PCL/PEG polymeric films prepared by a solvent-casting method from day 0 to day 30. Results represent mean ± SEM, *N* = 3. (D) Porosity of the PCL/PEG films measured at the beginning (day 0) and at the end (day 30) of the drug release experiment. Results represent mean ± SEM, N = 3. ***P < 0.001; ns, not significant. (E) Pore size distribution of the PCL/PEG films measured at the beginning (day 0) and at the end (day 30) of the drug release experiment. N = 3.

We next studied the pore size change on the surfaces of the films after the drug release experiment. Using SEM, there was a trend towards more pore formation as the percentage of PEG increased (Fig. 9A–B).

Due to the rapid leaching out of the PEG, 90 PCL:10 PEG, 80 PCL:20 PEG, 70 PCL:30 PEG, and 60 PCL:40 PEG films degraded by 6.6 ± 0.4 %, 14.2 ± 2.0 %, 30.0 ± 1.5 %, 38.8 ± 1.5 % and 53.6 ± 2.7 %, respectively, in three days and then reached a plateau. Besides, it should be noted that the weight difference between day 0 and day 30 was 54.6 % in the 50 PCL:50 PEG film (3 %), which is larger than the weight of the loaded PEG. This finding could be attributed to the increased porosity value, which could reduce the dead-end fraction in the film after the leaching out of the PEG, and also due to the increased contact area of the PCL and solution, which could enhance the PCL hydrolysis (Fig. 9C).

Besides, the porosity of the 70 PCL:30 PEG, 60 PCL:40 PEG and 50 PCL:50 PEG films increased significantly by 15.5 %, 17.4 % and 32.5 %, respectively, after the drug release experiment (Fig. 9D). Fig. 9E showed the changes in pore size distribution within the polymeric film before and after the drug release experiment. Compared to the original film, the 90 PCL:10 PEG (3 %), 80 PCL:20 PEG (3 %) and 70 PCL:30 PEG (3 %) films exhibited minimal or no change in pore size distribution. In contrast, the pore size distributions of the 60 PCL:40 PEG (3 %) and 50 PCL:50 PEG (3 %) films displayed a noticeable change, with the maximum pore size increasing from 6.5 to 13.7 μm^2^ and from 17.0 to 17.2 μm^2^, respectively.

### 3.9 Biocompatibility evaluation of the polymeric implant on FF and TM cells

The cell viability of human conjunctival fibroblasts (FF) and human trabecular meshwork (TM) cells was measured after 24 h of exposure to the media incubated with the drug solutions collected from the polymeric films with different compositions and at different time points. FF and TM cells did not show any significant decrease in cell viability, supporting the non-toxic and biocompatible characteristics of the polymeric films (Figs. S5 and S6).

## 4 Conclusion

Drug delivery implants can provide long-term drug release and directly deliver drugs to the targeted location. In this study, the aim was to develop a synergistic rate-retarding effect implant for monoclonal antibody release, and our work presents a novel approach by combining rate-controlled microporous membranes with drug-loaded PLGA microspheres. This innovative manufacturing method offers a simple but effective solution to the challenges associated with rapid burst drug release, while also providing broad flexibility in the development of customized biodegradable medical implants.

In this study, we have highlighted the critical importance of emulsifier concentration and osmotic pressure during PLGA microsphere fabrication, showcasing their impact on achieving a high encapsulation efficiency, minimal burst release, and sustained stability over time. Besides, a series of microporous membranes made of different FDA-approved polymers were developed. Our study demonstrated that, due to different polymer properties, membranes composed of a blend of hydrophobic and hydrophilic polymers exhibited superior control over pore formation and film degradation compared to membranes fabricated by blending two different hydrophobic and immiscible polymers. By elucidating the influence of various substitutions and concentrations on membrane permeation properties, we have enhanced our knowledge of tailored drug delivery applications, contributing to advancement in the field of membrane materials.

Furthermore, we monitored the drug release kinetics of the implants with optimized PLGA microspheres, different rate-controlling membrane formulations as well as different implant surface modifications. We found that the implant that was fabricated by 60 PCL: 40 PEG (3 %) polymer with 0.1 %*W*/*V* PLGA barrier exerted the best drug releasing profile and successfully released 83.6 % of IgG over 4 weeks. *In vitro* experiments showed that all the polymeric film formulations demonstrated good biocompatibility and no significant toxicity in both human trabecular meshwork cells and human conjunctival fibroblasts, suggesting their potential use in patients to prevent postoperative fibrosis following MIGS.

In summary, this study demonstrates the potential of polymer rate-controlled membrane reservoirs loaded with PLGA microspheres to sustained release monoclonal antibodies for 4 weeks. There are several areas for future improvements in this study. Firstly, the 50:50 PLGA used in this study degrades faster than other PLGA formulations with different lactide/glycolic acid molar ratios. Therefore, selecting alternative PLGA compositions could further optimize the drug release profile. Secondly, SEM offers insight into surface morphology, but additional techniques like Atomic Force Microscopy (AFM) could be employed to fully elucidate pore formation and interpenetrating structures within the polymeric films. Additionally, future work will concentrate on loading specific antibodies into the implant and assessing their impact on cellular function to better understand the effectiveness of this mAb delivery system. These improvements will further enhance the therapeutic effectiveness and translational potential of this drug delivery system.

## CRediT authorship contribution statement

**Mengqi Qin:** Writing – original draft, Software, Methodology, Formal analysis, Conceptualization. **Jinyuan Luo:** Writing – review & editing, Software, Methodology, Formal analysis. **Brihitejas Patel:** Writing – review & editing, Software, Methodology, Data curation. **Kai Xin Thong:** Writing – review & editing, Software, Methodology, Data curation. **Samar Latefa:** Writing – review & editing, Software, Formal analysis. **Daniel Shao:** Writing – review & editing, Software, Methodology, Data curation. **Alexander Tanner:** Writing – review & editing, Software, Methodology, Data curation. **Cynthia Yu-Wai-Man:** Writing – review & editing, Supervision, Resources, Funding acquisition, Conceptualization.

## Declaration of competing interest

The authors declare the following financial interests/personal relationships which may be considered as potential competing interests: Cynthia Yu-Wai-Man reports financial support was provided by 10.13039/100014013UKRI Medical Research Council. If there are other authors, they declare that they have no known competing financial interests or personal relationships that could have appeared to influence the work reported in this paper.

## Data Availability

No data was used for the research described in the article.

## Appendix A Supplementary data

Supplementary material Image 1
