# Supplementary material for: Developing a synergistic rate-retarding polymeric implant for controlling monoclonal antibody delivery in minimally invasive glaucoma surgery
Source: Int J Biol Macromol. 2024 Jun;272:132655. doi: 10.1016/j.ijbiomac.2024.132655 (PMC11780753; doi:10.1016/j.ijbiomac.2024.132655)
Supplement: Supplementary file 1 — Supplementary material [file mmc1.docx]

**Supplementary**

**Developing a synergistic rate-retarding polymeric implant for controlling monoclonal antibody delivery in minimally invasive glaucoma surgery**

### Mengqi Qin^1^, Jinyuan Luo^1^, Brihitejas Patel^1^, Kai Xin Thong^1^, Samar Latefa^1^, Daniel Shao^1^, Alexander Tanner^1^, Cynthia Yu-Wai-Man^1^

1. Faculty of Life Sciences & Medicine, King’s College London, London, SE1 1UL, UK.

**Corresponding Author:**

Cynthia Yu-Wai-Man, MBBS FRCOphth PhD

King’s College London

London SE1 1UL, UK

[cynthia.yu-wai-man@kcl.ac.uk](mailto:cynthia.yu-wai-man@kcl.ac.uk)

**Table S1.** Summary of the physiochemical properties for PLA, PCL and PLGA (50:50).

|  | **Polylactic acid** | **Polycaprolactone** | **Poly(lactic-co-glycolic acid)**  **(50:50)** |
| --- | --- | --- | --- |
| **Synonym** | PLA | PCL | PLGA |
| **Chemical Structure** | 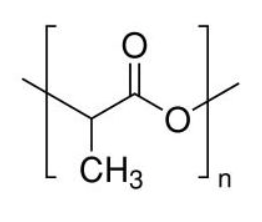 | 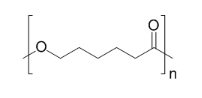 | 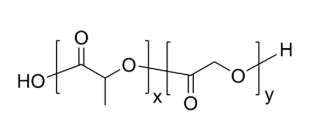 |
| **Linear Formula** | (C_3_H_4_O_2_)n | (C_6_H_10_O_2_)_n_ | (C_3_H_4_O_2_)x(C_2_H_2_O_2_)y |
| **Molecular Weight (Da)** | 30,000 | 50,000 | 10,000-20,000 |
| **Purity (%)** | >98 | ≤ 100 | 99.3 |
| **Melting Point (**°C) | 150-160 | 60 | 262 |
| **Intrinsic Viscosity (dL/g)** | 0.63 [1] | 1.07 [2] | 0.5-0.6 [3] |

**Table S2.** Detection in centrifuged supernatant from fresh plain MP4, fresh plain MP5, and lyophilized plain MP4 using Nanodrop at 280 nm absorbance.

| **Formulation** | **Detection Timepoint** | **Nanodrop Result (mg/mL)** |
| --- | --- | --- |
| Plain MP4 (fresh) | After Preparation | 0 |
|  | Day 1 | 0 |
| Plain MP5 (fresh) | After Preparation | 0 |
|  | Day1 | 0 |
| Plain MP4 (lyophilized) | Day1 | 0 |


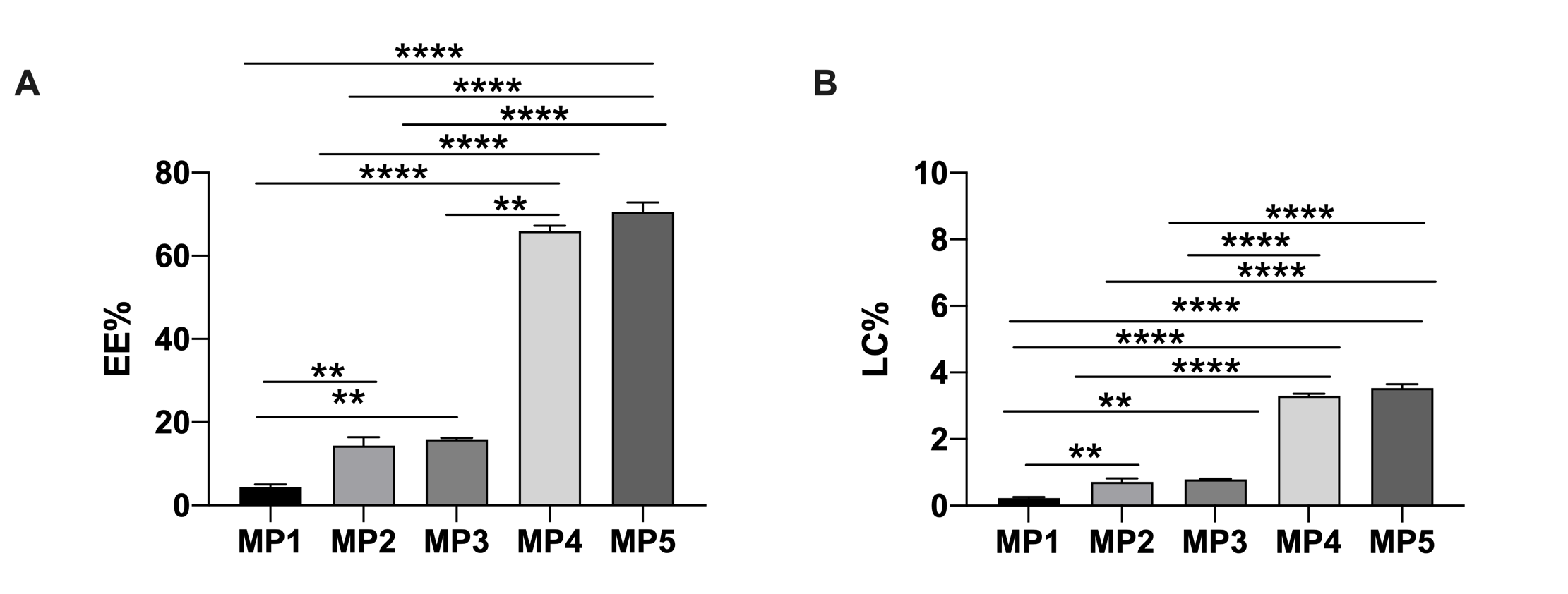
 **Figure S1:** Drug encapsulation efficacy (EE) and drug loading capacity (LC) of different microparticles were measured using the BCA assay. (A) Effect of different parameters on the drug encapsulation efficacy. (B) Effect of different parameters on the drug loading capacity.


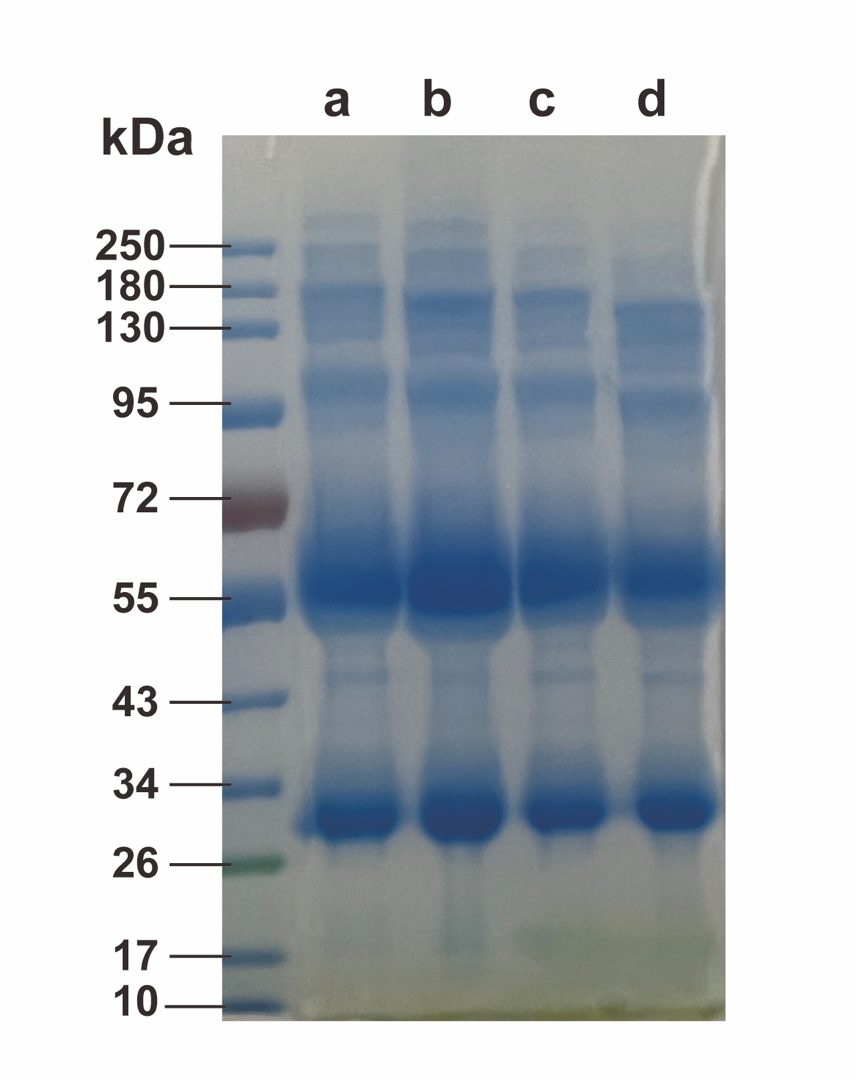


**Figure S2:** Coomassie-blue-stained SDS-PAGE gel for the drug release from 10 mg of PLGA microparticles. Sample collected from day 1 (lane a), sample collected from days 2-3 (lane b), sample collected from days 4-6 (lane c), and sample collected from days 7-40 (lane d). The protein markers with molecular masses 10, 17, 26, 34, 43, 55, 72, 95, 130, 180 and 250 kDa are shown.

**
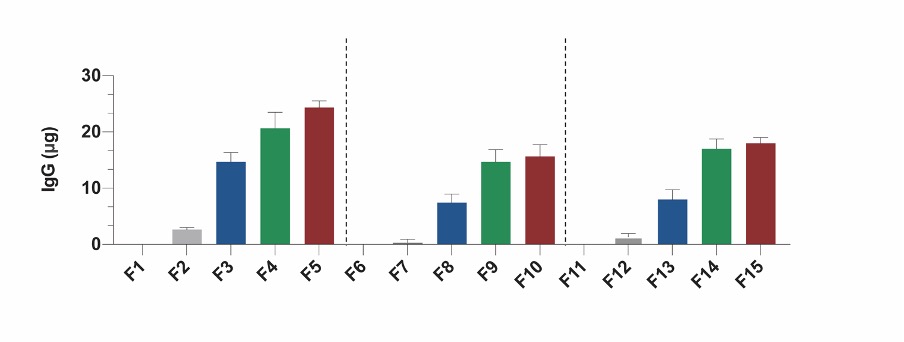
**

**Figure S3:** Cumulative amount of IgG released from F1-F15.


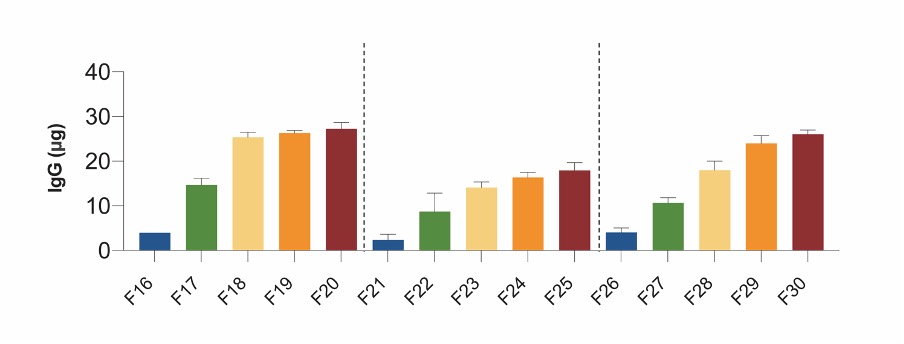


**Figure S4:** Cumulative amount of IgG released from F16-F30.


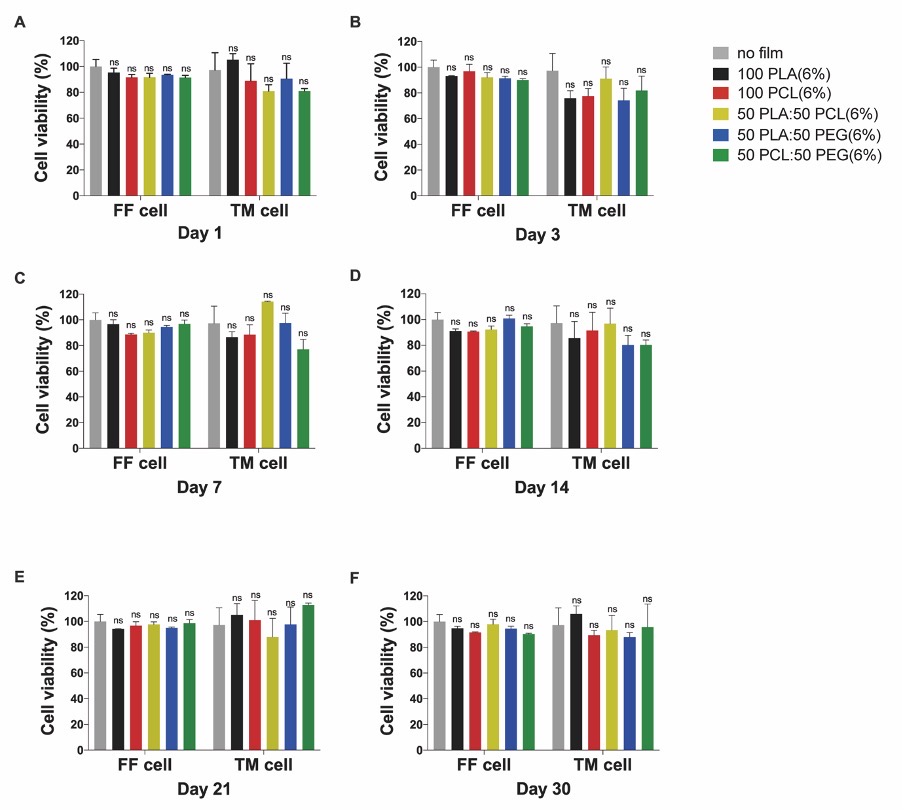


**Figure S5:** Cell viability of FF and TM cells was assessed after 1-day treatment with the no drug control or the incubation solutions of the polymeric film collected from (A) day 1, (B) day 3, (C) day 7, (D) day 14, (E) day 21, and (F) day 30. The values were normalized against untreated control cells. Results represent mean ± SEM, N = 3. ns, not significant.


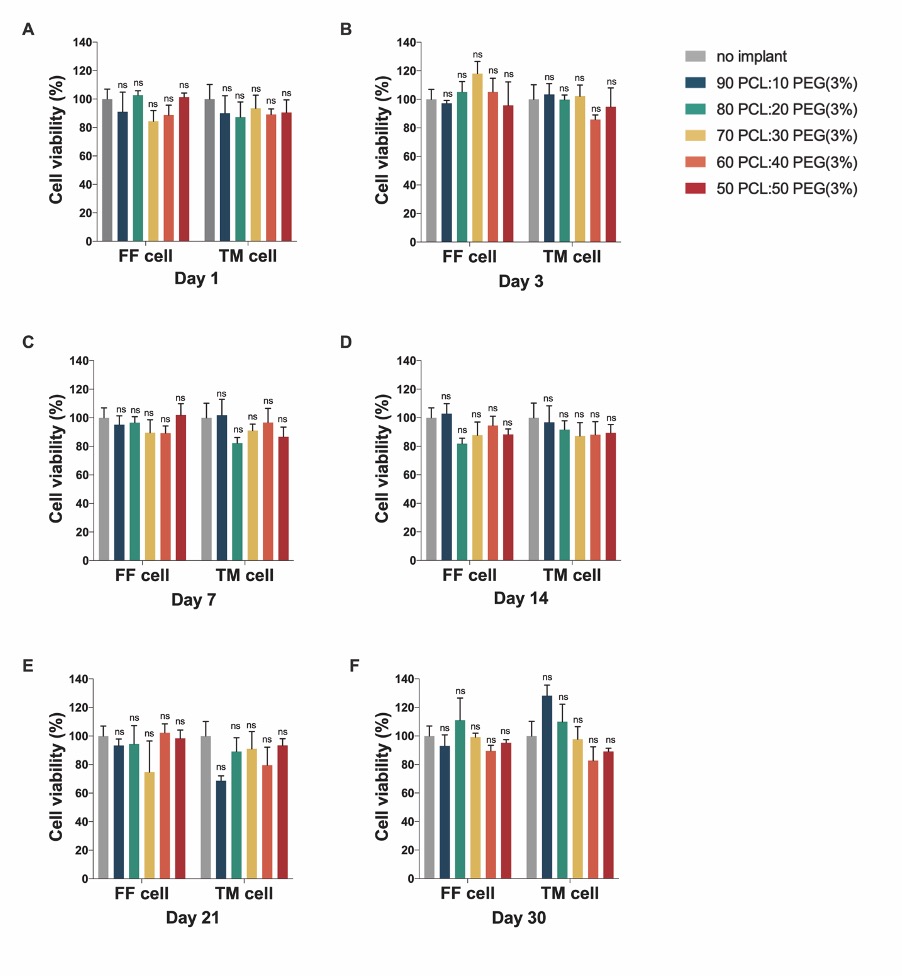


**Figure S6:** Cell viability of FF and TM cells was assessed after 1-day treatment with the no drug control or the incubation solutions of polymeric films with different ratios of PCL/PEG collected from (A) day 1, (B) day 3, (C) day 7, (D) day 14, (E) day 21, and (F) day 30. The values were normalized against untreated control cells. Results represent mean ± SEM, N = 3. ns, not significant.

**References**

[1] K. I. Ku Marsilla and C. J. R. Verbeek, Modification of poly(lactic acid) using itaconic anhydride by reactive extrusion, *European Polymer Journal,* vol. 67, pp. 213-223, 2015, doi: 10.1016/j.eurpolymj.2015.03.054.

[2] F. T. Yang, Y. M. Chen, and S. P. Rwei, Influence of Cross-Linking and Crystalline Morphology on the Shape-Memory Properties of PET/PEN/PCL Copolyesters Using Trimesic Acid and Glycerol, *Polymers (Basel),* vol. 15, no. 9, 2023, doi: 10.3390/polym15092082.

[3] E. Catiker, M. Gumusderelioglu, and A. Guner, Degradation of PLA, PLGA homo- and copolymers in the presence of serum albumin: a spectroscopic investigation, *Polymer International,* vol. 49, no. 7, pp. 728-734, 2000, doi: 10.1002/1097-0126(200007)49:7<728::Aid-pi443>3.0.Co;2-3.
